# Metabarcoding of native and invasive species in stomach contents of Great Lakes fishes

**DOI:** 10.1371/journal.pone.0236077

**Published:** 2020-08-11

**Authors:** Justin G. Mychek-Londer, Subba Rao Chaganti, Daniel D. Heath

**Affiliations:** 1 The Great Lakes Institute for Environmental Research (GLIER), University of Windsor, Windsor, Ontario, Canada; 2 Department of Integrative Biology, University of Windsor, Windsor, Ontario, Canada; King's College London, UNITED KINGDOM

## Abstract

As aquatic invasive species (AIS) proliferate worldwide, a better understanding of their roles in invaded habitats is needed to inform management and introduction prevention strategies and priorities. Metabarcoding of stomach content DNA (scDNA) shows considerable promise in such regard. We thus metabarcoded scDNA from two non-native fish species (alewife (*Alosa pseudoharengus*) and rainbow smelt (*Osmerus mordax*)), and three native ones (bloater (*Coregonus hoyi*), ninespine stickleback (*Pungitius pungitius*), and slimy sculpin (*Cottus cognatus*)). Fishes (N = 376) were sampled in spring 2009 and 2010 from 73–128 m depths at three Lake Michigan sites. Four mitochondrial cytochrome oxidase 1 (CO1) primer sets designed to target five potential AIS prey, and a universal aquatic invertebrate CO1 primer set targeting both native and AIS prey were used. Quality controlled prey amplicons were matched to three AIS prey: *Bythotrephes longimanus* (mean percent frequency occurrence, all samples = 7%), *Cercopagis pengoi* (5%), and *Dreissena rostriformis bugensis* (11%). Neither invasive prey *Dreissena polymorpha* nor *Hemimysis anomala* were detected. Native prey *Leptodiaptomus sicilis*, *Limnocalanus macrurus*, and *Mysis diluviana* were relatively common in scDNA (respective mean percent occurrences, all samples: 48%, 25%, 42%). Analysis of variation in prey occurrences for sample site, predator species, sample year, sample depth, and predator total length (TL) indicated site and predator species were most important. However, *B*. *longimanus* occurrence in scDNA depended upon predator TL, perhaps indicative of its unique defensive spine limiting susceptibility to predation until fishes exceed species-specific gape-based limitations. Our analysis of native and invasive prey species indicated possible indirect AIS impacts such as native predators switching their diet due to AIS-driven losses of preferred native prey. Metabarcoding demonstrated that AIS are integrated components of the offshore Lake Michigan food web, with both native and non-native predators, and both invasive and native prey are affecting species interactions across multiple trophic levels.

## Introduction

Analyses of aquatic food webs have widespread applications in conservation biology, ecosystem-based resource management, and in quantifying the roles and impacts of aquatic invasive species (AIS) [1–4]. AIS generally pose the second largest threat to aquatic biodiversity after habitat loss and disrupt food webs by causing species extinctions [3, 5, 6], inducing bottom-up and top-down trophic cascades in energy flow [7–9], outcompeting native species for limited prey resources [10], and by consuming larval or egg stages of native species impacting recruitment [11]. Thus, quantitative estimates of AIS roles in aquatic food webs are needed to allow fisheries managers and fish conservationists to objectively assess trophic impacts of AIS upon exploited and at risk species.

The Laurentian Great Lakes (hereafter Great Lakes) have experienced numerous species introductions and invasions post-European settlement. Furthermore, Great Lakes ecosystems are difficult effectively sample due to their size, depth, and community complexity. At least 186 non-native species have established in the Great Lakes, with widely varying ecosystem impacts [12–15]. For example, the alewife (*Alosa pseudoharengus*) has limited the natural recruitment of lake trout (*Salvelinus namayacush*) due to its predation on larvae [16, 17] and through its contributions as a prey fish to thiamine deficiency in adult predatory lake trout [17–19]. Likewise, due to its predation on larvae, rainbow smelt (*Osmerus mordax*) although an important prey species in the Great Lakes, also negatively impacts recruitment of native pelagic fishes [20, 21]. Resource competition among these fishes is difficult to demonstrate without measures of prey abundance; however, diet overlap among rainbow smelt, alewife, and native fishes has been noted as moderate to high in offshore Great Lakes regions [22]. Competition for some microcrustacean prey may also occur among rainbow smelt, alewife, and native fishes [23].

With a rapid increase in established AIS in the Great Lakes over past decades [24, 25], many AIS occupy a wide diversity of trophic levels and are important predators and prey [22, 26–28]. For example, native invertebrates commonly consumed by the non-native alewife and smelt, and the native fishes ninespine stickleback (*Pungitius pungitius)*, slimy sculpin (*Cottus cognatus*), and bloater (*Coregonus hoyi*) became extremely rare following predation by the AIS macroinvertebrate *Bythotrephes longimanus* in the Great Lakes [29–32]. However, *B*.*longimanus* is itself now a significant seasonal dietary component for some of the same predator species [28, 33]. Additional key AIS that have substantially impacted Great Lakes food webs include the zebra mussel (*Dreissena polymorpha*), quagga mussel (*D*. *rostriformis bugensis)*, a predatory waterflea (*Cercopagis pengoi*), and the bloody red mysid shrimp (*Hemimysis anomala*) [34, 35]. Quantifying the roles of these AIS in food webs can help determine management strategies for mitigating their impacts on Great Lakes basin ecosystems and native taxa.

Characterization of the roles of AIS in food webs has been conventionally performed through visual diet assessments [i.e., 36–38]. However, such methods can be biased by differential digestion rates and are time consuming [39–42]. Furthermore, cryptic prey species are difficult to identify even for well-trained taxonomists, especially when prey are partially digested [43, 44], reducing the identification potential for AIS and limiting potential early management responses [45–47]. Mitochondrial cytochrome oxidase subunit 1 [(CO1); 48–50] metabarcoding of multiple taxa or targeted species such as AIS from stomach content DNA (scDNA) can be helpful in addressing diet determination shortcomings [51, 52].

For example, Waraniak et al. [53] determined the diets of predatory fishes using 18S rDNA metabarcoding and showed significant seasonal shifts consistent with prey availability. Leray et al. [54] used a metabarcoding approach to describe diets of coral reef fishes, demonstrated highly complex food web interactions, and showed high levels of trophic partitioning among spatiotemporally overlapping fishes. Sensitivity to detect target prey species or groups of species using “universal” primer sets coupled with metabarcoding can be high [55, 56]. Furthermore, the sequence data itself permits accurate identification of the prey species, provided the prey barcode sequence is archived. While metabarcoding is powerful [e.g., 57], false negatives can result from factors including: stochastic PCR error, PCR inhibitors, amplification bias, within-species sequence variation, inadequate replication, and low overall or relative DNA concentration in mixed samples [58–60].

Although previous studies have examined diets using metabarcoding of scDNA to detect AIS, the Great Lakes, and especially hard-to-access offshore deepwater regions, are under-studied in these contexts. Thus, we sought to provide semi-quantitative information on roles of non-native and native predators and their native and invasive invertebrate prey occupying the offshore food web of Lake Michigan by using CO1 metabarcoding of predator fish scDNA. Our three objectives were to use metabarcoding of scDNA to: 1) determine presence/non-detection of target AIS prey, as well as for three abundant and ecologically important native prey species in diets; 2) determine if variation in diets existed across space and time; and 3) to assess whether specific abiotic and biotic factors affected the presence/non-detection of AIS and native prey in fish scDNA.

## Methods

### Field collections

**Fig 1:**
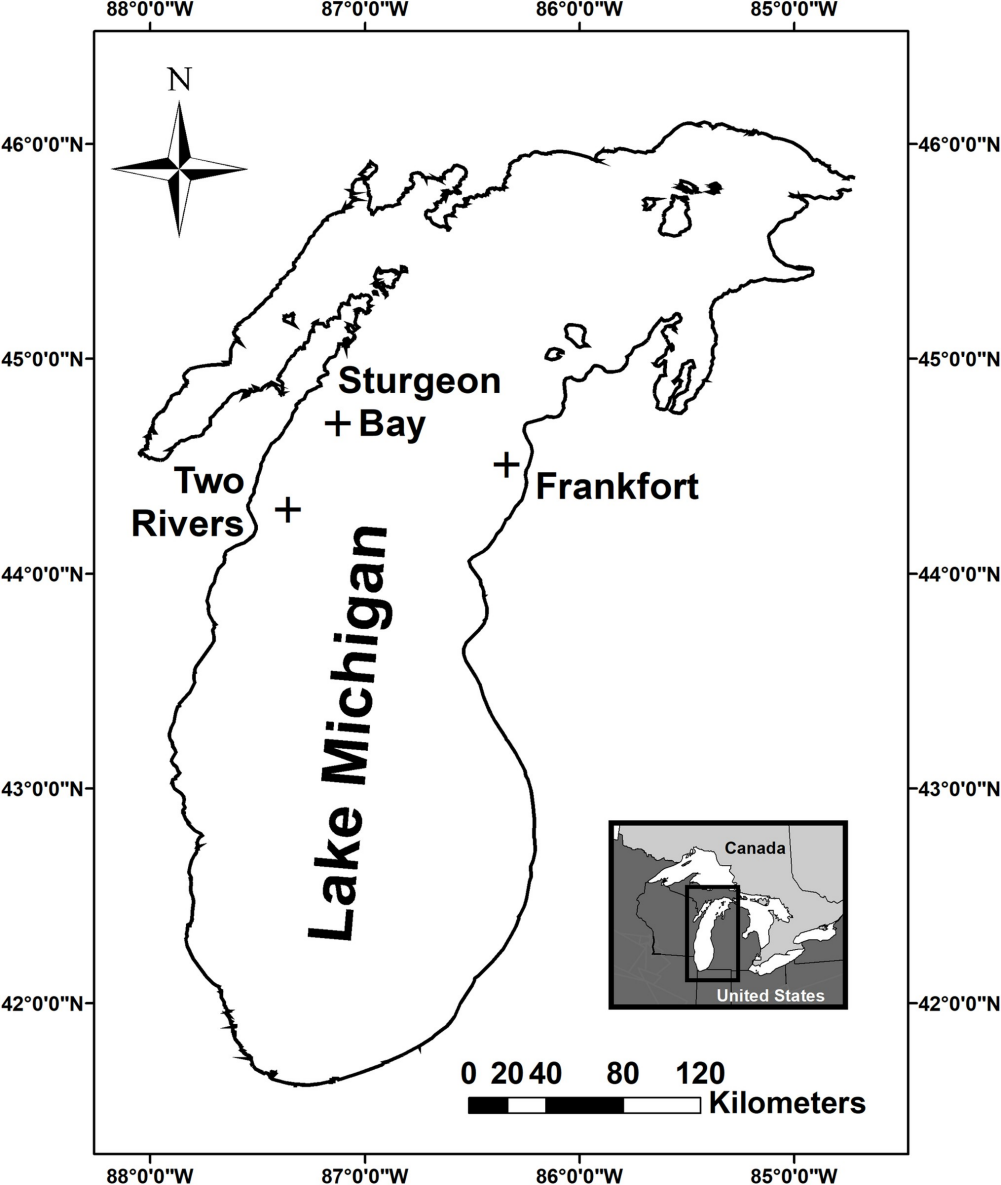
Sampling sites for the five predator fish species collected for mitochondrial cytochrome oxidase one (CO1) metabarcoding of stomach content DNA (scDNA). Single geo-located sampling sites are given. However, multiple local sites alongside these points were sampled to account for different depth strata or multiple tows at single depths. Depths included 73, 82, 91, 99, 110, and 128 m.

Alewife, rainbow smelt, ninespine stickleback, slimy sculpin, and bloater were sampled April 1 to 15, in 2009 and/or 2010 using bottom trawls for 5–10 minutes at three offshore Lake Michigan sites (Table 1; Fig 1). The USGS Great Lakes Science Center (GLSC) sampled fishes at sites offshore Frankfort, MI (44° 30′39′′N, 86 20′18′′W) and Sturgeon Bay, WI (44° 42′1′′N, 87 21′26′′W) using a 13 m Yankee trawl, as permitted by states of Michigan and Wisconsin. Commercial fishermen (Susie-Q: https://www.facebook.com/Susie-Q-Fish-Company-480212705346840; date last accessed June 24, 2020) were targeting rainbow smelt during the sampling period and provided this species as well as bycatch species offshore of Two Rivers, WI (44° 17′57′′N, 87 21′26′′W) using a 31 m otter trawl under approval of the State of Wisconsin. Trawl depths included 73, 82, 91, 99, 110, and 128 meters (Table 1). All collected fishes in a trawl, or a subsample of very large catches were immediately sorted by species. No endangered or protected species were involved in field studies. Up to 60 samples per species, per trawl, were subsampled and immediately frozen on board research vessels at -20°C for later scDNA recovery. Humane and ethical practices for euthanizing fishes during “intensive field sampling of large open aquatic habitats” used were approved by animal care and ethics committees of Michigan and Wisconsin in lieu of the permissions given for sampling [61; additional details: 22, 62].

**Table 1:** Collection details for zooplanktivorous fishes. Predator fish TL = total length (nearest millimeter (mm)).

Predator	Site	Year	Sample size	Mean TL (mm) ± SD
Alewife				
	Frankfort	2009	19	112.6 ± 30.6
	Frankfort	2010	20	103.3 ± 31.6
	Sturgeon Bay	2010	20	80.2 ± 8.2
	Two Rivers	2010	20	124.9 ± 28.5
Rainbow smelt				
	Frankfort	2009	20	72.6 ± 31.0
	Frankfort	2010	20	104.8 ± 16.1
	Sturgeon Bay	2010	20	117.0 ± 15.0
	Two Rivers	2010	20	101.3 ± 4.5
Ninespine stickleback				
	Frankfort	2009	19	68.7 ± 7.2
	Frankfort	2010	20	68.2 ± 5.7
	Sturgeon Bay	2010	20	69.7 ± 6.5
	Two Rivers	2010	20	66.3 ± 5.3
Slimy sculpin				
	Frankfort	2009	19	72.0 ± 10.7
	Sturgeon Bay	2009	20	67.2 ± 13.6
	Two Rivers	2009	20	75.1 ± 11.0
Bloater				
	Frankfort	2009	19	146.2 ± 50.4
	Frankfort	2010	20	162.6 ± 30.9
	Sturgeon Bay	2010	20	120.1 ± 15.8
	Two Rivers	2010	20	135.2 ± 24.5

All samples were from Julian Days of the Year from 101–112 (April 1–15). N = 376 total samples. Because of limited sample availability, fishes at Frankfort were subsampled from three to five depth strata (including 73, 82, 91, 110, and 128 meters (m)). Sturgeon Bay and Two Rivers fishes were sampled from single depths of 82 and 99 m respectively. Fish predator species names: Alewife = *Alosa pseudoharengus*, Rainbow smelt = *Osmerus mordax*, Ninespine stickleback = *Pungitius pungitius*, Slimy sculpin *= Cottus cognatus*, and Bloater = *Coregonus hoyi*.

### Stomach content sampling

Fish were thawed, weighed, and measured to total length (TL, nearest 1.0 millimeter (mm)). Whole stomachs (esophagus to just below pyloric valve) were excised and individually preserved each in 95% ethanol until further processing of stomach contents. To achieve desired sample sizes for each species, subsamples of fishes at each site and in each year were selected based upon stratified random sampling for body size and capture depth. Overall, the aim was for a balanced design and representation of diets from all sizes available per fish species (Table 1).

At medium to high magnification using fine forceps, conspicuous prey in bolus form or otherwise easily distinguishable prey and prey parts were removed from each stomach and placed in 1.7 mL microcentrifuge tubes for DNA digestion. Next, small soft-tipped paintbrushes were used to loosen potential remaining prey tissue, while minimizing predator DNA release. Recovered prey tissues were added to each 1.7 mL tube. All prey contents from individual diet samples were used, except for the largest stomach content samples from the largest sized alewife and bloater. These samples required mixing of prey contents to a homogeneous level in a Petri dish followed by sub-sampling of a portion of the homogenous prey mix into respective 1.7 mL tubes for DNA digestion. Sub-sampling was necessary as wet prey content for these large samples exceeded 0.60 mL, the target maximum starting volume for DNA extraction. After transferring prey tissues, 95% ethanol was added to submerge the tissues, and contents were vortexed for 30 seconds at high speed.

### Extraction of scDNA

Tubes with the stomach contents in ethanol were centrifuged at 13,000 x g for 15 minutes at 4°C. Supernatant ethanol was discarded and a Eppendorf 5301 VacuFuge Centrifugal Vacuum Concentrator was used to dry stomach contents in each tube. Immediately after drying the stomach contents 200–500 μL of 1.0 mm glass mill beads (BioSpec Products) and 600–900 μL of digestion buffer (5.84 g NaCL · L^-1^, Tris-HCl pH 8.0 final concentration 50 mM, EDTA pH 8.0 final concentration 10 mM, SDS to final concentration of 0.5%,diluted in ddH_2_O) were added, depending upon starting volumes of materials present to digest. A Mini-Beadbeater-24 (Fisher Scientific LTD., BioSpec.) was used and set at 50 strokes per second to homogenize prey in tubes for 40 seconds. This was followed by 60 seconds of cooling tubes on ice, repeated 3–8 times based both upon starting material volumes and visual inspection of sample homogeneity.

After homogenizing stomach content samples, 8 μL of Proteinase-K (20 mg · mL^-1^), was added. Then, samples were digested at 38°C with gentle rocking for 8–12 hours. Next, scDNA was extracted from 150 μL of the supernatant using a Tecan Freedom EVO 150 Liquid Handling System, a carboxylate magnetic bead-based protocol, three ethanol washes, and an elution of extracted scDNA into 150 μL of 1X TE. Lastly, extracted scDNA samples were sealed and frozen at -20°C until further use.

### Polymerase chain reactions

Five polymerase chain reaction (PCR) primer sets targeting a conserved sub-portion of the 658 bp mitochondrial gene cytochrome oxidase one (CO1) barcoding region were used to amplify prey DNA sequences. Four primer sets were designed, and used to target five specific AIS including: 1) *B*. *longimanus*; 2) *C*. *pengoi;* 3) *H*. *anomala*; and 4) *D*. *rostriformis bugensis* and *D*. *polymorpha* [see: 63 for primer design details; S1 Table]. The fifth was a universal CO1 primer set designed for aquatic invertebrates [64] used previously for scDNA from nearshore species of Lake Erie fishes [65] and which was used to target native and AIS of invertebrate prey.

A three-step PCR approach was used for the target-specific AIS primer sets. A two-step approach was used for the universal primer set, where round one PCRs included a 5' extended sequence tag on each primer. Second-round PCRs for the target AIS primer sets used the same protocols as did first-round PCRs, but used cleaned and concentrated DNA from first-round PCR product reactions to increase detection sensitivity. This second-round of PCRs was not needed for universal primer set PCR amplicons as most scDNA samples successfully amplified by visual assessment after first-round PCRs. Third-round PCRs for the target-specific AIS primer sets and second-round PCRs for the universal primer set were a short-cycle PCR designed to ligate the sample identification sequence barcode and high throughput sequencing (HTS) adaptor sequences for HTS library preparation.

Total reaction volume for a single PCR for each of the five primer sets in first-round PCRs was 25 μL and consisted of: 2.5 μL of 10X Taq reaction buffer (Bio Basic, Cat. #37A); 0.5 μL each of 10 μM forward and reverse primers; 0.1 μL of Taq polymerase at 5 units · μL^-1^ (Bio Basic, Cat. #HTD0078); 1.0 μL of 10 μM dNTPs; 3.5 μL of 20 mM MgSO_4_ (Bio Basic Cat. #37B); 0.2 μL of 20 μg · μL^-1^ bovine serum albumin (BSA); 1.0 μL of extracted scDNA; and the remaining total volume was nuclease free Milli-Q water. Thermal cycling protocols for all first-round PCRs consisted of: an initial denaturation cycle at 94°C for 2 minutes; followed by 35 cycles of: a) denaturation at 94°C for 45 seconds; b) annealing at 59°C for 30 seconds; and c) extension at 72°C for 45 seconds. After the 35 cycles there was a final single cycle of extension at 72°C for 10 minutes followed by a 4°C hold. Two positive and negative (blank) controls were used for all PCRs. Positive controls used DNA extracted from known species samples for the five target AIS whereas negative controls lacked any additions of DNA. PCR amplification was visually assessed by agarose gel electrophoresis.

To maximize detection of AIS, PCR products from all first-round PCRs were used in additional individual second-round of PCRs for the target AIS PCR primer sets. These second-round PCRs followed the same protocol for the first-round PCRs but used first-round PCR products as the template (see above). Only one round of PCRs were needed for the universal primer set as many scDNA samples amplified successfully based on band detection on agarose gels. The final second-round PCR products from target AIS primer sets and the final first-round PCRs from the universal primer set were combined for individual scDNA samples based on relative amplification strength to help ensure even sequencing depth. Each scDNA sample amplicon mix was cleaned using a magnetic bead protocol, which removed small amplified fragments and primer dimers less than approximately 100 base pairs. Sera-Mag Speed Beads (GE Healthcare Life Sciences) were used following the protocol for the Agencourt AMPure XP PCR Purification Beads. Subsamples of cleaned PCR products were run on agarose gels to confirm reduction or removal of dimers and amplicons below 100 bp and the retention of amplified bands of larger size. Products were stored at -20°C.

### Sequencing library preparation

The final short-cycle PCR was designed to ligate a unique sequencing barcode and the HTS adaptor sequences to the PCR amplicons for each sample. These PCRs consisted of 2.5 μL of 10X Taq reaction buffer, 25 mM MgCl_2_, 0.2 mM of each dNTP, 0.5 μM forward primer + Uni-B adaptor, 0.5 μM reverse primer plus Uni-A adaptor, 0.1 units Taq polymerase, 10 μL of cleaned PCR product, and remainder of ddH_2_O for a total reaction volume of 25.0 μL. Short-cycle PCR began with a 2-minute denaturation at 95°C followed by 6 cycles of 95°C denaturation for 30 seconds, 60°C annealing temperature for 30 seconds, 72°C extension for 30 seconds and a final single extension at 72°C for 5 minutes. Subsamples from each primer set were checked using gel-electrophoresis and UV imaging to confirm small increases in fragment sizes indicative of successful ligation of the barcode and adaptor sequences.

All ligated PCR products were combined in equal proportions and gel extracted based upon the expected sized band from the mixture. Duplicate gel extracts were obtained using a GenCatch^TM^ Advanced Gel Extraction Kit (Epoch Life Science Inc.) followed by elution into a final volume of 20 μL. The inclusion of target species primer set amplicons through these steps for sequencing was to verify that the agarose bands present after first or second-round PCRs were the intended target AIS. Measures of presence and non-detection from target AIS primer sets were used in favor of quantitative measures using sequence read numbers as the target AIS primer sets were not validated as being truly species-specific.

Gel-extracted barcoded amplicons were analyzed in duplicate using an Agilent High Sensitivity DNA chip on an Agilent 2100 Bioanalyzer (Agilent Technologies, Germany) to determine final amplicon concentration and check that distributions of fragments at high abundances were the size ranges expected. Each sample replicate was next diluted to a final concentration of 60.0 pmol · μL^-1^ and replicates were combined in equal proportions into a single sample. The final barcoded meta-sample was sequenced on a 318-chip on the Ion Torrent System (Life Technologies, USA).

### Sequence analyses

The Quantitative Insights Into Microbial Ecology (QIIME) software pipeline [66] was used to remove sequenced amplicons with average quality scores < 19.0 and to remove amplified fragments < 100/150 bp for target AIS primer and universal primer sets, respectively. All sequences with > three primer-template mismatches were also removed. To verify amplification of target AIS from target species primer set PCRs, sequences resulting from our target AIS primer sets were compared to files containing all variations of AIS specific reference CO1sequences downloaded from GenBank and Barcode of Life [67, 68]. Universal primer set sequences were BLASTed against a custom reference database containing sequences for: 1) the five selected target AIS; and 2) sequences for three important native invertebrate prey species (*Leptodiaptomus sicilis*, *Limnocalanus macrurus*, and *Mysis diluviana*). The three native species were selected as we expected them to be common diet items in both native and non-native predators [37, 69] and because they are likely to interact with the target AIS directly or indirectly [70, 71]. Additionally, these three native invertebrate species have been reported as invasive or as posing invasion risks outside the Great Lakes [72, 73]. BLAST analyses against the custom databases were performed using the *map reads to reference* function in QIIME with default parameters, except having adjusted the minimum match value to 98% for reads from the universal primer set, and having set this value to 96% as a minimum match value for reads from any of the target AIS primer sets.

### Presence/non-detection analyses

Assessments of the occurrence (presence/non-detection) of specific prey species in each predator scDNA sample were completed. Presence data for the target AIS primer sets was based on positive gel image results for individual scDNA samples; however, a requirement was that all presence scoring ultimately be confirmed by a match with the NGS sequence data for each such sample. Additionally, another requirement was at least three sequences per scDNA sample of given prey species to count the prey species as present to avoid sequencing artifacts driving up positive presence scoring.

For the universal primer set data, a minimum of 250 high quality sequences per sample were required for the sample to be included in analyses. The minimum sequence number threshold was also set to three matched sequences for target AIS to be identified in individual predator scDNA samples using the universal primer set. If the threshold number of sequences was not met, the prey species was scored as not detected for the individual scDNA sample. The binary presence/non-detection data for target AIS prey were used in further analyses, described below, for testing for biotic and abiotic effects on the prevalence of the target AIS in Lake Michigan planktivore diets.

Sequence data from the universal primer set PCRs were also used to characterize the role of the three selected native crustacean macroinvertebrate species in the diet of predator fishes. For analysis of the three native prey, the requirements to be counted as present included: 1) at least three sequences of the native prey species were present per sample and; 2) the sequences had a relative read abundance (RRA) ≥ 0.10%, relative to the total number of quality controlled sequences recovered from that sample [see: 74]. For informational purposes, RRA values were summed for these three native prey in each predator. Lastly, the summed RRA percentage for each predator was averaged across the entire data set of 376 scDNA samples.

### Statistical analyses

The influences of biotic and abiotic factors on observed occurrences of the target AIS and three selected native prey species were assessed using binary (present/not detected) logistic Generalized Linear Models (GLMs). Main effects included predator species, sampling year, and sampling site as fixed effect categorical independent variables. Sampling depth and predator TL were included as continuous covariates. Subjects were the individual fish predators (coded as one or zero for prey present or not detected, respectively) and statistical models were run for each prey species separately. The number of maximum iterations was increased to 10,000 in the modeling procedure and all other default settings were applied in SPSS (IBM Corp. Released 2011, Version 20.0) for each GLM.

## Results

### Target AIS primer sets

**Fig 2:**
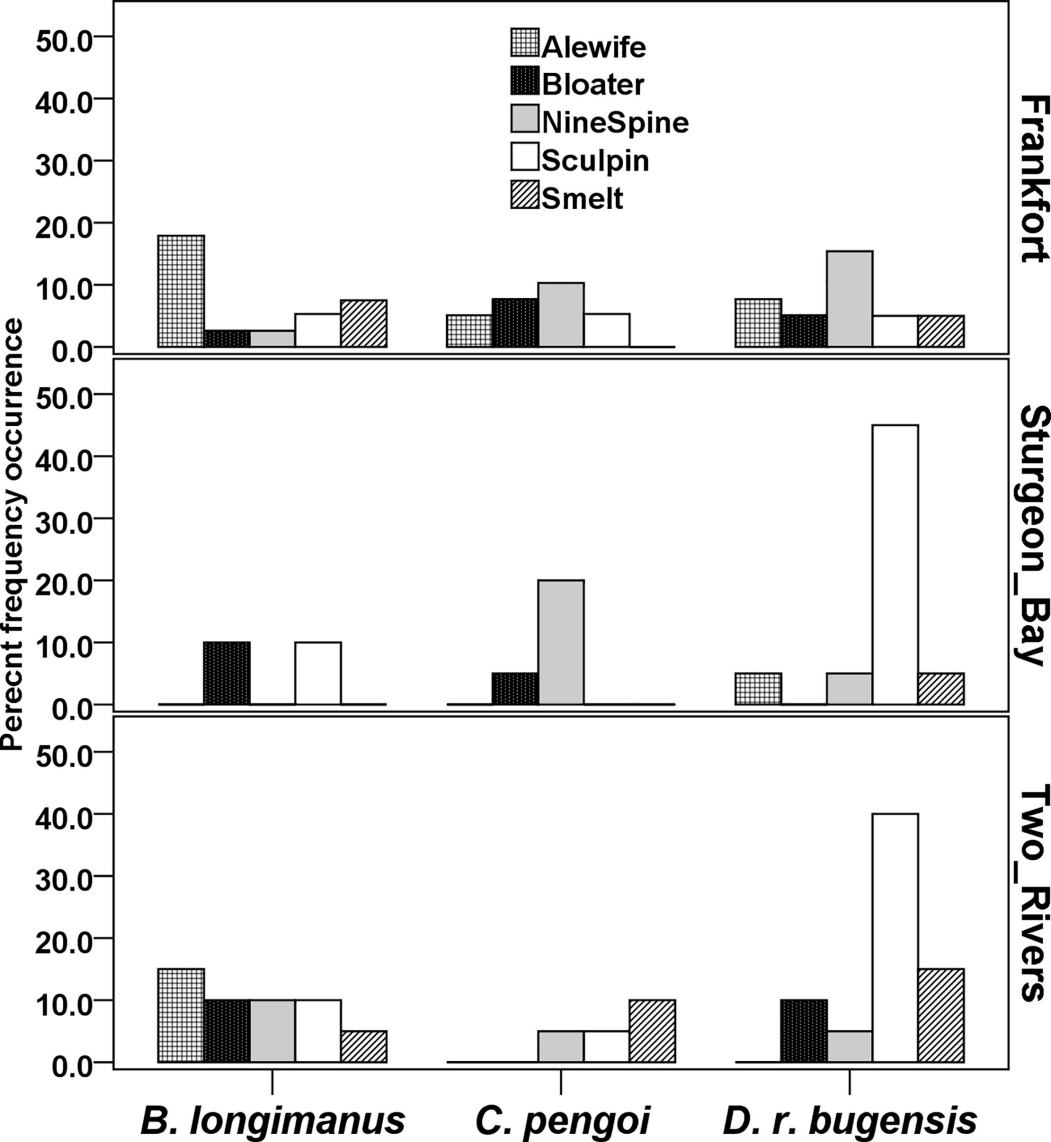
Percent frequency of occurrence of target AIS in predator scDNA samples across all predator fish samples, including from multiple years and depths at each site. The three prey on the x-axis, are named fully as *Bythotrephes longimanus*, *Cercopagis pengoi*, and for Dreissenid only includes *Dreissena rostriformis bugensis*. No positive results were observed for *Dreissena polymorpha*, or *Hemimysis anomala*, thus these prey were excluded in results for this figure. Fish predator species names: Alewife = *Alosa pseudoharengus*, rainbow smelt = *Osmerus mordax*, ninespine stickleback = *Pungitius pungitius*, slimy sculpin = *Cottus cognatus*, and bloater = *Coregonus hoyi*. Sample sites are denoted on the right-side y-axis.

Second-round PCR gel imaging revealed 26, 47, and 10 scDNA samples with positive results for the *C*. *pengoi*, *Dreissena* spp., and *H*. *anomala* target primer sets respectively (N = 376 total samples). No positive results from the *B*. *longimanus* target primer set were observed after first or second-round PCRs. Of the 376 samples, target species primer set sequence data confirmed the presence of *C*. *pengoi* in the diets of 19 of 26 PCR-identified predators (sequence read number in positive scDNA samples ranged from 3 to 585; Table 2; Fig 2) and confirmed *D*. *rostriformis bugensis* in 40 of 47 positively PCR-identified scDNA samples (sequence read number in positive scDNA samples ranged from 1 to 3440; Table 2; Fig 2). No matches for *D*. *polymorpha* resulted from the *Dreissena* spp. based target-specific primer set sequence data and no matches resulted from the target-specific primer set sequence data for target AIS *H*. *anomala*.

**Table 2:** Percent frequency of occurrence of target AIS in predator diets (N = 376) at each site determined using CO1 metabarcoding of scDNA.

		Predator sample size (N)	*Bythotrephes longimanus*		*Cercopagis Pengoi*		*Dreissena rostriformis bugensis*	
Site	Predator		TSPS	UPS	TSPS	UPS	TSPS	UPS
Frankfort	Alewife	39	0.0	17.9	5.1	0.0	7.7	0.0
	Smelt	40	0.0	7.5	0.0	0.0	5.0	2.5
	Stickleback	39	0.0	2.6	10.3	0.0	15.4	2.5
	Sculpin	19	0.0	5.3	5.3	0.0	5.0	0.0
	Bloater	39	0.0	2.6	7.7	0.0	5.1	0.0
Sturgeon	Alewife	20	0.0	0.0	0.0	0.0	5.0	0.0
Bay	Smelt	20	0.0	0.0	0.0	0.0	5.0	0.0
	Stickleback	20	0.0	0.0	20.0	0.0	5.0	0.0
	Sculpin	20	0.0	10.0	0.0	0.0	45.0	5.0
	Bloater	20	0.0	10.0	5.0	0.0	0.0	0.0
Two Rivers	Alewife	20	0.0	15.0	0.0	0.0	0.0	0.0
	Smelt	20	0.0	5.0	10.0	0.0	15.0	5.0
	Stickleback	20	0.0	10.0	5.0	0.0	5.0	5.0
	Sculpin	20	0.0	10.0	5.0	0.0	40.0	0.0
	Bloater	20	0.0	10.0	0.0	0.0	10.0	0.0

Frankfort data are for both years and all depths combined. TSPS = results from target AIS specific primer sets. UPS = results from the universal primer set. Results for TSPS were based upon positive results from PCRs and gel imaging and required confirmation with sequencing results. All UPS results were based upon sequencing data, and for *D*. *rostriformis bugensis* positive results from this primer set were found in some of the same individual fishes for which positive results from the respective target primer set occurred. *Dreissena polymorpha* and *Hemimysis anomala* were excluded as all respective results for target AIS specific and universal primer sets were negative. Fish predator species names: alewife = *Alosa pseudoharengus*, rainbow smelt = *Osmerus mordax*, ninespine stickleback = *Pungitius pungitius*, slimy sculpin = *Cottus cognatus*, and bloater = *Coregonus hoyi*.

### Universal primer set

Across the 376 scDNA samples, the average number of reads per sample using the universal primer set was 7,041.6 (min = 0, max = 140,500). Of these, 70 had < 100 sequence reads, 111 had < 250, 136 < than 500, and 180 scDNA samples had < 1000 sequence reads per sample after filtering for sequence length and quality, leaving 196 which had > 1000 reads after filtering (also see reference # 63, and S1 File for additional information on sequence reads). Although no positive hits resulted for *B*. *longimanus* using the target-specific primer set (see above), 27 scDNA samples were positive for *B*. *longimanus* using the universal primer set with a mean sequence read number per positive sample of 316.5 (± SD 566.3; min = 3, max = 1687; Table 2; Fig 2).Of the predator species sampled, alewife from Frankfort had the highest occurrences of *B*. *longimanus* in their scDNA; had this prey in at least one sample of those from four of the five depth strata there, and consumed *B*. *longimanus* in both 2009 and 2010. The second highest mean occurrence for a predator species of *B*. *longimanus* sequences in scDNA was in alewife sampled at Two Rivers.

The universal primer set also amplified *D*. *rostriformis bugensis* sequences, but comparatively, the *Dreissena* spp. target-specific primer set had much greater sensitivity (see results above). In total, the universal primer set produced 22 quality-filtered *D*. *rostriformis bugensis* sequences in five scDNA samples (Table 2). AIS sequences for *C*. *pengoi*, *D*. *polymorpha*, or *H*. *anomala* were not detected using the universal primer set.

**Fig 3:**
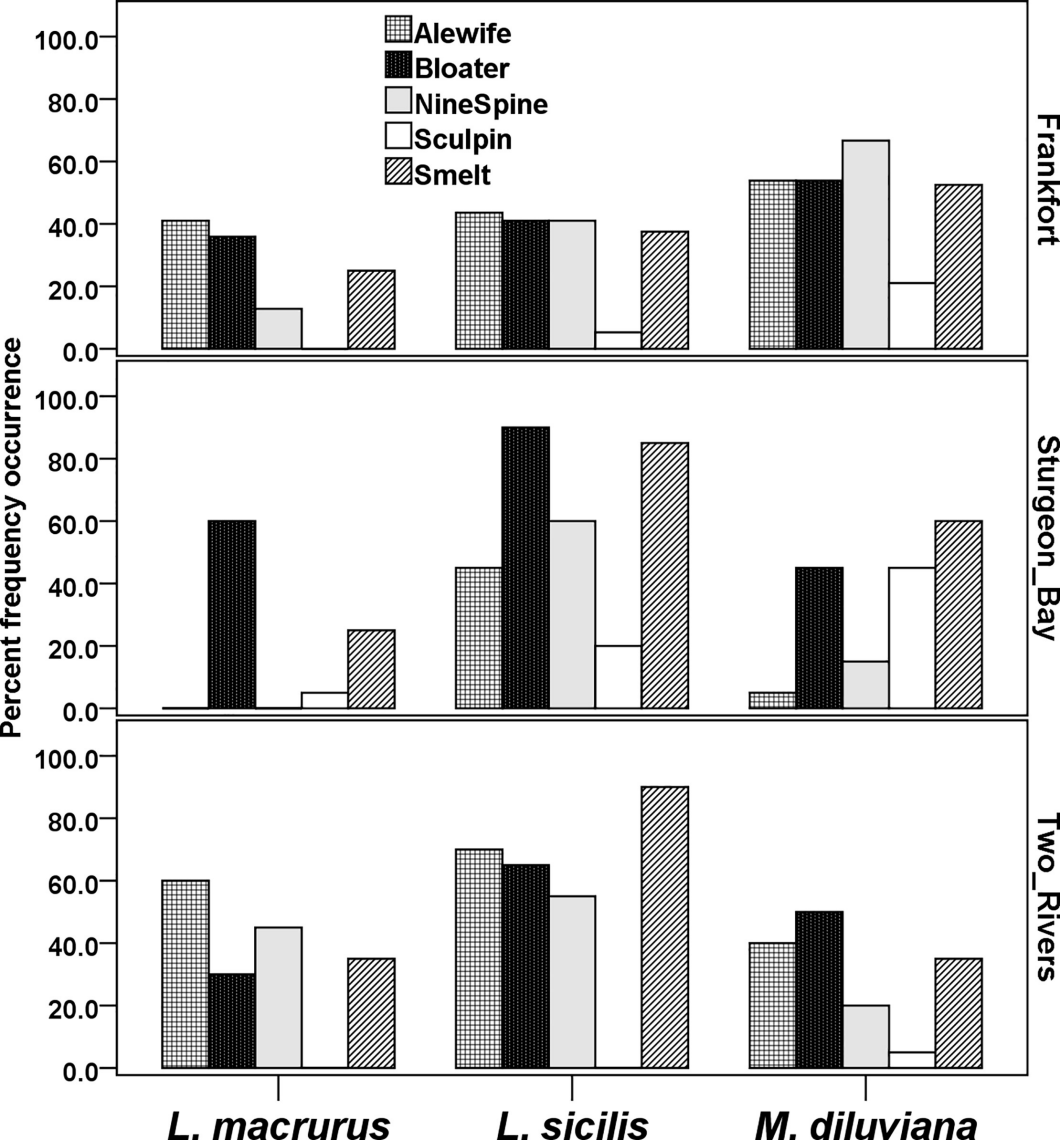
Percent frequency of occurrence of three native prey in predator scDNA samples across all predator fish samples, including from multiple years and depths at each site. All sequences in this data figure were produced by the universal primer set. Fish predator species names: alewife = *Alosa pseudoharengus*, rainbow smelt = *Osmerus mordax*, ninespine stickleback = *Pungitius pungitius*, slimy sculpin = *Cottus cognatus*, and bloater = *Coregonus hoyi*. On the x-axis full invertebrate prey species are named as: *Limnocalanus macrurus*, *Leptodiaptomus sicilis*, and *Mysis diluviana*. Sample sites are on the right-side y-axis.

As expected, frequencies of occurrences for the three native prey were substantially higher than AIS in scDNA samples. For example, four predator species had > 35% overall mean occurrences of *L*. *sicilis*, four predator species had > 50% occurrence of *M*. *diluviana* in scDNA for Frankfort samples, and four predator species sampled at Two Rivers had *L*. *macrurus* occurrences between 30–60% (Table 2, Fig 3). Detection sequences for each of these three native prey occurred in 48.3% (*L*. *sicilis)*, 41.7% (*M*. *diluviana*), and 25.5% (*L*. *macrurus)* of all the 376 scDNA samples in the dataset. An overall mean RRA of 35.1% of all quality-controlled reads produced by the universal primer set resulted from the three target native prey species.

### Presence non-detection modeling

**Fig 4:**
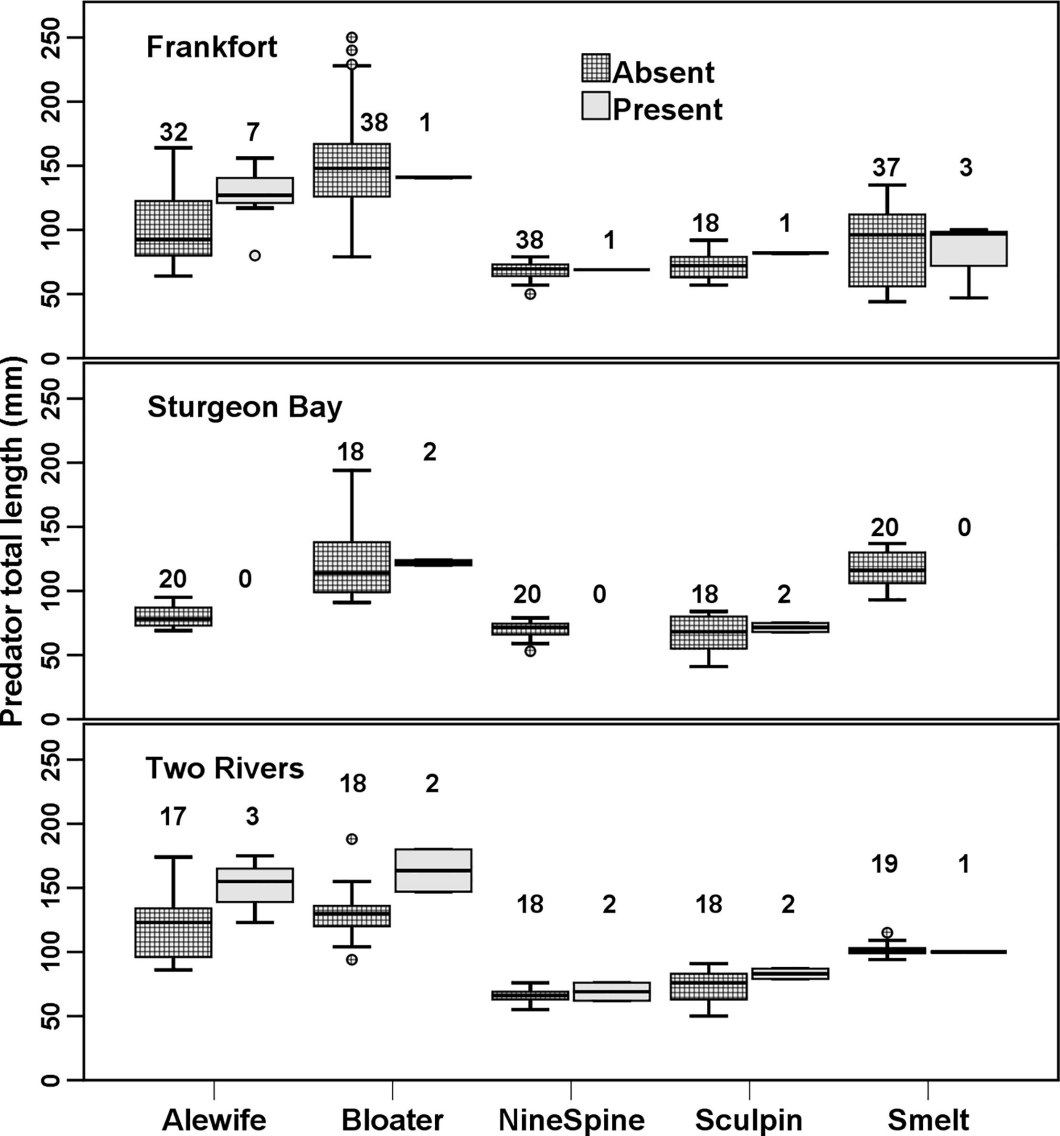
Total lengths (TL) of predator fishes with and with without AIS prey *B*. *longimanus* in scDNA. Data across all sampled depths and years at each site were combined for analysis. One-way ANOVA tests did not reveal any significant differences in mean TL values for comparisons within each predator species among each sample site (N = 15; all p > 0.050). Dots above or below boxplot whiskers indicate individual outlier samples with low or high TL values. Fish predator species names: alewife = *Alosa pseudoharengus*, rainbow smelt = *Osmerus mordax*, ninespine stickleback = *Pungitius pungitius*, slimy sculpin = *Cottus cognatus*, and bloater = *Coregonus hoyi*.

Presence/non-detection GLM for the AIS *B*. *longimanus* showed significant predator species total length effects (Table 3; Fig 4). The GLM for *C*. *pengoi* yielded no significant effects. The *D*. *rostriformis bugensis* GLM showed a significant predator species effect, but no significant effects for any other variable (Table 3). GLMs for the three native prey *L*. *sicilis*, *L*. *macrurus*, and *M*. *diluviana* each revealed significant effects in each model for both predator species and for site (Table 4). The data set and corresponding meristics used for these statistical analyses and other assessments in our manuscript are provided within S1 File.

**Table 3:** Results from the binary logistic generalized linear model occurrence analyses for the three target AIS prey detected.

AIS prey, model term	Wald Chi-Square	Degrees freedom	Significance
*B*. *longimanus*			
Intercept	3.597	1	0.058
Predator	5.361	4	0.252
Site	2.422	2	0.298
Year	0.905	1	0.341
Depth	0.842	1	0.359
Predator total length	3.901	1	**0.048**
*C*. *pengoi*			
Intercept	7.733	1	**0.005**
Predator	6.190	4	0.185
Site	0.587	2	0.746
Year	0.233	1	0.629
Depth	0.795	1	0.373
Predator total length	0.067	1	0.796
*D*. *rostriformis bugensis*			
Intercept	1.464	1	0.226
Predator	10.872	4	**0.028**
Site	0.856	2	0.652
Year	0.012	1	0.914
Depth	1.268	1	0.260
Predator total length	2.751	1	0.097

Effects of fish predator species, sampling site, depth, year of sample, and predator total length (TL, nearest millimeter) were tested using quality controlled sequences. Subjects were each individual fish predator scDNA sample. Each model was tested one prey type at a time. The dependent variable was the assigned a value of zero or one for non-detection or presence (respectively) of the prey of interest. Main effects included predator species, year, and sampling site as fixed effect categorical independent variables. Depth and predator TL were included as continuous covariates in each model for each prey type. Significant values are in bold in the rightmost column. AIS prey named fully as *Bythotrephes longimanus*, *Cercopagis pengoi*, and *Dreissena rostriformis bugensis*.

**Table 4:** Binary logistic generalized linear model presence/non-detection analyses for three native prey.

Native prey, model term	Wald Chi-Square	Degrees freedom	Significance
*Leptodiaptomus sicilis*			
Intercept	0.448	1	0.503
Predator	21.012	4	**0.000**
Site	13.133	2	**0.001**
Year	2.002	1	0.157
Depth	0.027	1	0.869
Predator total length	0.200	1	0.655
*Limnocalanus macrurus*			
Intercept	3.261	1	0.071
Predator	11.774	4	**0.019**
Site	6.415	2	**0.040**
Year	0.327	1	0.567
Depth	0.086	1	0.465
Predator total length	0.535	1	0.770
*Mysis diluviana*			
Intercept	0.091	1	0.762
Predator	9.598	4	**0.048**
Site	6.228	2	**0.044**
Year	2.669	1	0.102
Depth	0.767	1	0.381
Predator total length	1.258	1	0.262

Predator species, sampling site, depth, year of sample, and predator total length were tested using quality-controlled sequences amplified by the universal primer set. Subjects were each individual fish predator. Each model was tested one prey species at a time. The dependent variable was the assigned value of zero or one for non-detection or presence of the prey of interest. Main effects included predator species, year, and sampling site as fixed effect categorical independent variables. Depth and predator TL were included as continuous covariates in each model for each prey type. Significant values are in bold in the rightmost column.

## Discussion

CO1 metabarcoding of native and non-native zooplanktivore fish scDNA revealed AIS prey that are known to have impacted Great Lakes food webs [13, 14, 16] and are among the “world’s worst 100 invasive species” [75]. Specifically, AIS *B*. *longimanus*, *C*. *pengoi*, and *D*. *rostriformis bugensis* were detected in multiple predator scDNA samples. AIS *D*. *polymorpha* or *H*. *anomala* were not detected in scDNA, despite using sensitive PCR primer sets designed for each species [63]. Metabarcodinging analysis of the three native prey in predator scDNA contributes to understanding the Lake Michigan offshore food web and exhibited frequencies of occurrences similar to published conventional diet analyses [i.e., 22, 37, 69]. Outside the Great Lakes, the three native prey identified in scDNA are of conservation concern [72, 73, 76, 77]. Sample site, predator species, and predator TL significantly influenced AIS and native prey occurrence patterns. Thus, especially when used as a tool in combination with other approaches, metabarcoding scDNA can help characterize food webs, particularly for large, hard-to-access ecosystems such as offshore Lake Michigan, where understanding dynamic, localized and potentially interacting influences from AIS impacts and changes in native species are critical to further conservation efforts.

Patterns of occurrence of AIS *B*. *longimanus* in scDNA may reflect aspects of its adaptive capacity, morphology, life history, and reproductive strategy. For example, its cyclical parthenogenesis and lack of over-winter adult survival coupled with over-wintering resting eggs, which can survive passage in gut-tracts of predator fishes [78], can result in strong patterns of seasonal prey availability [28]. However, spring-summer hatches may be delayed in cold, deep areas such as we sampled [28, 79] making consumption patterns of *B*. *longimanus* by predators in such offshore zones unpredictable. Resting eggs and prey body parts re-suspended into the water column, or unexpected hatching during spring turnover, could have increased the detection of *B*. *longimanus* [78]. Up to three-month earlier than expected onsets of population growth, six-month longer durations in occurrences, and unexpected survival of over wintering adult female *B*. *longimanus* have been observed for some other large lakes, exemplifying its adaptive capacity [79–81]. Typically, Great Lakes *B*. *longimanus* do not survive over winter [82, 83], and major swarms from emergent diapausing hatching eggs usually appear the following summer, [i.e., 28, 78]. Predator TL significantly affected *B*. *longimanus* presence in scDNA of some predator species, perhaps due to the defensive distal tail-spine that limits susceptibility to predation until fishes reach a minimal gape size [84–88; see Fig 4]. This would also serve to help explain how fish predator TL was a significant factor, but fish predator species was not.

No biotic or abiotic factors were associated with presence of AIS prey *C*. *pengoi* in our sampled predator scDNA. Life history and reproductive strategies for *C*. *pengoi* are similar to those of *B*. *longimanus*, and likely influenced predation patterns [89, 90]. Lack of significant predator TL effects on occurrence patterns of *C*. *pengoi* in scDNA may reflect that it is smaller than *B*. *longimanus*; however, its similar, albeit smaller, defensive spine has been cited as a factor limiting its consumption as prey by smaller fishes [91]. Although *C*. *pengoi* has been reported at occurring at higher densities further from shore in large lakes [92], no significant effects of site or depth sampled were found with respect to its presence in scDNA. *C*. *pengoi* was previously found in fish stomachs sampled in the spring from deep, offshore areas of Lakes Michigan and Huron [93], supporting our positive detections in scDNA. Our data will help better characterize the spatiotemporal overlap of *C*. *pengoi* and its predators and dynamics between them, which are especially important for hard-to-access deepwater offshore Lake Michigan food webs.

Non-detection of *D*. *polymorpha* in all scDNA samples agrees with its general absence in deep offshore zones of the Great Lakes [8, 94–98]. However, the related species *D*. *rostriformis bugensis* was identified in scDNA from fish samples for almost every combination of our five predator species and three sample sites. As very few conventional diet studies on our predator species have reported dreissenids, or even their shells, predators likely consumed veligers or recently settled juvenile non-shelled *D*. *rostriformis bugensis*. Microscopic pelagic veligers remain planktonic for three to four weeks, during which time mortality rates, including from predation, can exceed 99% [99]. Additionally, lack of recognition of digested early stage *D*. *rostriformis bugensis* may have limited its detection in conventional diet analyses in slimy sculpin [see: 37]. Slimy sculpin also uniquely lack a swim bladder and is a benthic species, resulting in possible greater predator-prey spatial overlap with *D*. *rostriformis bugensis*. High *D*. *rostriformis bugensis* occurrences in slimy sculpin scDNA could have also resulted from incidental (non-selective) consumption during predation of preferred benthic taxa such as *Diporeia hoyi* or chironomids [e.g., 37]. An intriguing possibility for high occurrences of *D*. *rostriformis bugensis* in scDNA for all predators in our study may be secondary prey detection due to the sensitivity of our methods. For example, the common native species *M*. *diluviana* consumes *Dreissena* spp. veligers [100, 101] and is a common preferred prey for all predators in our study [22, 102, this study]. Therefore, although scDNA metabarcoding is highly sensitive and cost-effective, we caution that considerations of life stage and incidental or secondary consumption of this prey may bias interpretations and influence conclusions.

The high mean overall relative quality-filtered sequence read abundance (RRA) for the selected native prey species (*L*. *sicilis*, *L*. *macrurus*, and *M*. *diluviana*) in the scDNA metabarcode library demonstrated that a few key native prey taxa can play an important role in diet composition across a diverse set of predator species, despite taxonomically diverse stomach contents. Sampling site was a highly significant factor for predation patterns on all three native invertebrate prey species, consistent with previous diet studies of offshore Great Lakes predator fishes [22, 37, 69]. For example, *M*. *diluviana* had the highest occurrences in scDNA at Frankfort, compared to our other sample sites, in agreement with relatively very high visually determined occurrences of this prey at that location, lending support to our results [i.e., 22, 37]. An increased reliance upon *M*. *diluviana* by the Lake Michigan (and other Great Lakes) predator fishes has occurred in recent decades because of the disappearance of the preferred native prey, *D*. *hoyi* [i.e., 103–105]. Furthermore, *D*. *hoyi* was consumed frequently at Sturgeon Bay and Two Rivers, but not for Frankfort sampled fish diets from a visual-based diet study with similarly derived stomach content samples [see: 22, 37]. Thus, the finding that sample site was an important predictor of *M*. *diluviana* occurrence in scDNA is not surprising. The pattern of native prey occurrence using scDNA metabarcoding analyses likely reflects ongoing AIS-induced changes in ecological processes (e.g., prey preference) in the food web, and our results are comparable with conventional diet studies for the same predator species [i.e., 37].

Significant differences were noted among predator species in their exploitation of the two native copepods *L*. *sicilis* and *L*. *macrurus*, and of the native mysid *M*. *diluviana*. This may be due to site-based differences in prey availability and predator-prey demand, perhaps resulting from AIS induced food web changes. For example, elevated consumption of native copepods *L*. *sicilis* and *L*. *macrurus* at Frankfort likely contributed to significant effects for sample site and predator species for these prey. This agrees with reported 1995–2005 diet trends indicating an increased reliance on *L*. *sicilis* and *L*. *macrurus* as *D*. *hoyi* declined [22]. Slimy sculpin had in some cases unexpectedly low occurrences of *L*. *sicili*s, *L*. *macrurus*, and *M*. *diluviana* in their diets, as slimy sculpin taken from the same trawl hauls as in our study had much higher occurrences of these prey based upon visual assessments [37]. The anomalously low occurrences of of these prey in slimy sculpin scDNA may have resulted from low sequence read numbers in individual samples that did not pass our quality filtering requirements. However, there was substantial variation among levels of occurrences of of these three prey among the other predators as well, except at Frankfort where they were present at similar levels of occurrences in predator species. The importance of both sampling site and predator species in determining prey occurrences in a study such as this is perhaps not surprising, as the native and non-native predator fishes sampled are able to adapt their predation tactics to dynamic food web conditions [i.e., 22, 37].

An expectation that non-native fish species should have consumed more AIS prey than native fishes was not generally supported. Lake Michigan alewife have been documented to have some of the highest reported proportions of *B*. *longimanus* as prey in summer and fall, when this prey is abundant [22, 28]. In our study, non-native alewife had the overall highest mean occurrence of *B*. *longimanus* in scDNA of all predator species, but did not consume it at Sturgeon Bay, one of our three sample sites, whereas the native fishes bloater and slimy sculpin did. In contrast, native ninespine stickleback had the highest mean occurrences of AIS *C*. *pengoi* in scDNA at two of three sites versus every other combination of predator and site. Further, one in five stickleback from Sturgeon Bay contained *C*. *pengoi*, while all non-native predators sampled there lacked it. Although published studies identified *B*. *longimanus* and *C*. *pengoi* in conventional diet studies [22, 28], less research has characterized them as being consumed by offshore Great Lakes fishes, especially by ninespine stickleback [22, 102]. Thus, predator species is an important consideration in the design and analyses of metabarcoding scDNA studies to detect and characterize AIS in food webs. However, it appears specializations of individual predator species or localized food web conditions were more important in influencing prey occurrence patterns than was predator endemism.

In summary, CO1 metabarcoding identified AIS and native invertebrate prey species in scDNA from two non-native and three native zooplanktivorous fish species sampled during spring from three offshore Lake Michigan sites at various depths, targeting five invertebrate AIS prey and detecting three. Analyses of the influences of biotic and abiotic factors upon AIS and native prey occurrences in scDNA indicated site-based ecological variation was the most important explanatory factor, followed by predator species, and predator TL. Native prey had expectedly higher levels of mean occurrences in predator scDNA than did AIS in the hard-to-access deepwater offshore Lake Michigan benthopelagic food web, but AIS were not uncommon. This may be due to the ecosystem accommodating AIS over time, and both native and non-native predators habituating to AIS as prey, as well as due to the high sensitivity of metabarcoding. Additionally, patterns of prey occurrences in scDNA appeared to reflect localized food web conditions at sites having experienced differential AIS impacts and corresponding predator-specific abilities to respond to ecological change. For example, at sites where *D*. *hoyi* abundance was low (due to AIS effects), increased predation on the remaining native and AIS prey could have resulted in elevated diet overlap among native and non-native predators. In conclusion, our scDNA metabarcoding approach provides a useful template for exploring the interactions among predators and prey in complex ecosystems and food webs. Further, scDNA metabarcoding data may help to objectively assign priorities for the prevention of the further spread of AIS to minimize the impacts on native species through trophic interactions.

## Supporting information

S1 Table AIS PCR primer set details (modified from Mychek-Londer JG, 2018. Ph.D. Dissertation). (DOC)

S1 File MS Excel worksheet for data and statistical analyses. (XLSX)
