# Supplementary material for: Metabarcoding of native and invasive species in stomach contents of Great Lakes fishes
Source: PLoS One. 2020 Aug 11;15(8):e0236077. doi: 10.1371/journal.pone.0236077 (PMC7419000; doi:10.1371/journal.pone.0236077)
Supplement: S1 Table — (DOC) [file pone.0236077.s001.doc]

| Prey species | Primer direction | Primer sequence 5’→3' | Number base pairs in amplified fragment |
| --- | --- | --- | --- |
| *Bythotrephes* | F | acctgcctgccgGCTGAGTTGGGACAGGCAGGG | 279 |
| *longimanus* | R | acgccaccgagcTGCTCCACTCTCTACGGCCCC |  |
|  |  |  |  |
| *Cercopagis* | F | acctgcctgccgGGGCCCCTGATATGGCTTTCCC | 338 |
| *pengoi* | R | acgccaccgagcGGCTGTGATTCCAACAGCTCAAACA |  |
|  |  |  |  |
| *Dreissena rostriformis bugensis/ Dreissena* | F | acctgcctgccgAGCATTGTTAAGGCACCGGCT | 295 |
| *polymorpha* | R | acgccaccgagcAGGGCGGATTTGGTGGGGGT |  |
|  |  |  |  |
| *Hemimysis* | F | acctgcctgccgTTGGGTCAGCCCGGTAGGTT | 283 |
| *anomala* | R | acgccaccgagcTCCACCCCGTACCAACCCCC |  |

Amplified fragment sizes include the COI target sequence, forward (F) and reverse (R) primers and include procedural sequencing adapters (forward adapter 5'→3' = acctgcctgccg; reverse adapter 5'→3' = acgccaccgagc).
